# On-demand cesarean section: assessing trends and socioeconomic disparities

**DOI:** 10.11606/S1518-8787.2019053001466

**Published:** 2019-12-23

**Authors:** Kharen Carlotto, Luana Patrícia Marmitt, Juraci Almeida Cesar

**Affiliations:** I Universidade Federal do Rio Grande. Faculdade de Medicina. Programa de Pós-graduação em Ciências da Saúde. Rio Grande, RS, Brasil; II Universidade Federal do Rio Grande. Faculdade de Medicina. Programa de Pós-graduação em Saúde Pública. Rio Grande, RS, Brasil

**Keywords:** Cesarean section, Women's health, Health Inequality Monitoring

## Abstract

**OBJECTIVE:**

to measure prevalence, evaluate trends and identify socioeconomic differences of on-demand cesarean section in the municipality of Rio Grande (RS), extreme south of Brazil, in 2007, 2010, 2013 and 2016.

**METHODS:**

all the puerperae residing in this municipality who had cesarean deliveries in one of the only two local maternity hospitals in the period 01/01-31/12 of the aforementioned years were part of this transversal study. Puerperae were interviewed using a single, standardized questionnaire at the hospital within 48 hours after delivery. The outcome was assessed based on the mothers’ report that the cesarean section was performed according to their request. The analysis consisted of the observation of the outcome's frequency in each year and the evaluation of its prevalence throughout this period through the chi-square linear trend test. Socioeconomic inequalities were assessed based on household income and women's schooling using the Slope Index of Inequality and the Relative Index of Inequality.

**RESULTS:**

In these four years, 5,721 cesarean deliveries were recorded among mothers living in this municipality (1,309 in 2007, 1,341 in 2010, 1,626 in 2013 and 1,445 in 2016). In this period, the rate of on-demand cesarean sections increased by 107%, from 10.5% (95%CI: 8.9% -12.2%) of the deliveries in 2007 to 21.7% (95%CI: 19.5% -23.8%) in 2016. This increase was more evident among those with lower household income and schooling level. Absolute inequality also increased, especially regarding schooling, while relative inequality sharply declined when assessed by household income.

**CONCLUSIONS:**

The increased on-demand cesarean sections in the study location is unsettling, despite the decreasing gap between extreme categories as a consequence of higher levels of this procedure among women of lower income and worse schooling.

## INTRODUCTION

In Brazil, 55% of all births were by cesarean section in 2016^1^. This rate is about fourfold the recommended by the World Health Organization (WHO) and at least twofold the observed in developed countries^2^.

The excess of this intervention involves not only clinical needs, but also non-obstetric factors such as convenience for the physician and, especially, the mother's desire. The mother's deliberate and assumed decision to perform this procedure, disregarding clinical indications to her or the baby is called on-demand cesarean section. The maternal reasons for this surgical event are fear of childbirth, interference with future sexual performance and lack of pain during labor^3^^,^^4^.

On-demand cesarean sections already account for 6% to 17% of all cesarean deliveries in the world^4^. The mothers’ request has been pointed out as one of the causes of the increasing number of this type of delivery^5^. In Brazil, there are few publications on this subject, especially when considering population-based studies. Only two studies were identified, one of them, performed in Rio Grande (RS) in 2007, showed a 11% rate of on-demand cesarean section^6^, similar to the 11.2% rate found in the hospital-based study *Nascer no Brasil: Pesquisa Nacional sobre Parto e Nascimento* (Birth in Brazil: A Nacional Survey into Labor and Birth), from 2011-2012^3^.

Cesarean section causes unnecessary risks to the mother such as infections, hemorrhage, anesthetic complications and higher mortality, and to the child, the main risk refers to neonatal respiratory morbidity^7^. It is not yet clear if on-demand c-section increases the incidence of prematurity and low birth weight^7^.

In general, cesarean sections are more frequent in women with lower obstetric risk, who deliver in the private sector and have a better education level and a more favorable economic situation^8^. Whether on-demand cesarean section varies significantly according to socioeconomic status is little known, as this disparity was not approached by Brazilian studies. These studies also did not investigate the prevalence of on-demand cesarean sections over the years^3^^,^^6^.

Our study aimed to evaluate the trend and socioeconomic gaps in the performance of on-demand cesarean sections in Rio Grande (RS), Brazil, in 2007, 2010, 2013 and 2016, considering the relevance of this theme for maternal and child health, as it involves the use of health services, the rights of its users and proper medical praxis, and for the opportunity to first-handily cover this theme using four population-based surveys in Brazil, also considering inequalities.

## METHODS

Our study is part of a larger project entitled “Perinatal Study” that evaluates the trend of indicators of pregnancy and childbirth care in Rio Grande (RS), a municipality with 215,000 inhabitants in the extreme south of the state of Rio Grande do Sul, Brazil. Four perinatal surveys using the same methodology were conducted in 2007, 2010, 2013 and 2016. We used a cross-sectional, population-based design in all these surveys.

All puerperae living in the municipality with deliveries in the only two local maternity hospitals in the period 01/01–31/12 of each survey year were included in these studies. Moreover, their newborn should have reached at least 500 grams and/or 20 weeks of gestational age. In this analysis, only those parturients who had a cesarean delivery were included, since the outcome was the event of on-demand cesarean section, namely, a cesarean section performed following the parturient request.

We used information from the four surveys (2007, 2010, 2013 and 2016) to estimate the sample size of the on-demand cesarean section prevalence. The outcome prevalence ranged from 10.5% (95%CI: 8.9%–12.2%) to 21.7% (95%CI: 19.5%–23.8%) in the study period. These parameters allowed us to work with a 1.9 percentage points margin of error and a 95% confidence level, totaling 1,533 puerperae. We could work with a 1.0 percentage point margin of error when considering a 17.2% overall prevalence of on-demand cesarean section (in the four surveys) and the available “n” of 5,721 puerperae with a 95% confidence level. All estimates were performed using the Epi Info 7.0^9^ program.

The information from surveys was collected through a questionnaire applied to the mothers within 48 hours after delivery. In each year of the study, four interviewers were selected and trained to apply the questionnaires. At the end of the training, a pilot study was conducted in the month before the beginning of data collection in each maternity unit. The interviewers identified pregnant women both from the rural and urban areas on the maternity hospital register on a daily basis and visited the infirmaries. The mothers answered the questionnaire only after agreeing to participate in the study, fully understanding and signing the informed consent form. The questionnaire gathered information on demographic, socioeconomic and reproductive characteristics, life habits, gestational morbidity, anthropometric data and the type of care the mothers received during pregnancy and delivery. In addition, information from the Pregnant Women's Card used for prenatal appointments and submitted on admission was copied in a standard form.

All questionnaires were printed and pre-coded, except for the latest survey. In 2016, interviews were conducted using a tablet with the Research Electronic Data Capture (REDCap)^10^ application. At the end of each working day, the interviewer coded the printed questionnaires and delivered them to the study site to be reviewed and sent for entry. Data were double entered in the free software Epidata 3.1^11^, by independent typists, students of *Programas de Iniciação Científica* (scientific initiation programs) of the Universidade Federal do Rio Grande (FURG). Regarding electronic questionnaires, data were uploaded daily to FURG's server and reviewed by one of the study supervisors.

Any discrepancy in the questionnaires was corrected and the respondent contacted again if necessary. The information was stored in a database to create derivative variables and for subsequent analyses after the conclusion of the consistency analysis. These steps were performed using the statistical package Stata version 13^12^.

The statistical analysis consisted first of the description of independent variables studied: maternal age, skin color, household monthly income, schooling, number of appointments and type of prenatal service (public or private), and childbirth financing type (public or private). The chi-square linear trend test for proportions was used to evaluate the prevalence trend of on-demand cesarean section in the study years (2007–2016). In the evaluation of disparity, outcome prevalence measurements were obtained according to parturient income and schooling. In this case, the chi-square linear trend test was also used.

The absolute and relative socioeconomic inequalities were assessed using the Slope Index of Inequality (SII) and the Relative Index of Inequality (RII)^13^. SII was obtained from regression of the outcome at the midpoints of relative income and schooling, resulting in values between zero and 1. To estimate relative income and schooling, the categories of these variables were initially set in ascending order to estimate the midpoint of distribution for each category. SII expresses, in percentage points, the absolute difference of the regression regarding the event of on-demand cesarean section between the midpoint of the lower level category (lower household income or worse schooling) in relation to the midpoint of the highest level category (higher household income or better schooling).

Logistic regression and the same classification for income and schooling were also used to estimate the RII, which were inserted as independent variables in the regression model. The regression coefficient and the standard error were used to estimate the odds ratio (OR) with a 95% confidence interval. This OR was, thus, the RII. The results were expressed as the relationship between the highest and the lowest category of income and schooling, reflecting the relative inequality based on the probability of individuals achieving the outcome. Thus, the greater the SII and/or RII, the greater the level of inequality in the socioeconomic hierarchy^13^.

About 7% of telephone interviews were retaken for quality control, with key questions from the original questionnaire to enable identification of inconsistencies. The agreement between responses of the two moments was verified through the Kappa Index, which ranged from 0.69 to 0.99 in the study years, revealing at least satisfactory agreement.

The Health Research Ethics Committee (CEPAS) of the Universidade Federal do Rio Grande approved the research protocol for each year of study under opinions 23116.5369/6.58 (2007), 23116.006258/2009-63 (2010), 23116.002623/2012-67 (2013) and 030-2015 (2016).

## RESULTS

According to the *Sistema de Informações sobre Nascidos Vivos* (SINASC – Live Birth Information System), 10,423 women in Rio Grande had at least one child in one of the local maternities in 2007, 2010, 2013 or 2016. Of this total, 10,242 were interviewed, which is equivalent to a 98.3% response rate. Over half of them (55.9% or 5,721) underwent cesarean section.

Table 1 shows the distribution of all puerperae according to the type of cesarean section performed, per survey and for several of the characteristics studied. The performance of on-demand cesarean sections was much higher among women aged 25 years and over (70%), white (80%), with a monthly income above two minimum wages (63%), with over 11 years of schooling (77%), who attended six or more prenatal appointments (96%) in the private system (88%), in which delivery also occurred (83%). The on-demand cesarean section rate in the four surveys was 17.2% (95%CI: 16.2–18.2).

**Table 1:** Characteristics of parturients who underwent cesarean section in Rio Grande, RS, Brazil, in 2007, 2010, 2013 and 2016.

Characteristic		Cesarean		Total	p-value*
		On-demand	Other		
Survey year					< 0.001
	2007	14.0%	24.7%	22.9%	
	2010	24.8%	23.2%	23.4%	
	2013	29.4%	28.2%	28.4%	
	2016	31.8%	23.9%	25.3%	
Age (full years)					< 0.001
	11-19	9.2%	14.6%	13.7%	
	20-24	20.6%	26.0%	25.1%	
	25-29	31.5%	25.3%	26.4%	
	30 and over	38.7%	34.1%	34.8%	
	Mean (standard deviation)	27.8 (5.9)	26.8 (6.6)	27 (6.5)	
Self-reported skin color					< 0.001
	White	79.8%	69.8%	71.5%	
	Brown	14.9%	20.0%	19.1%	
	Black	5.3%	10.2%	9.4%	
Household monthly income in minimum wages (n=5.663)					0.001
	1 (lower)	14.8%	28.8%	26.4%	
	2	28.0%	36.8%	35.3%	
	3 (higher)	57.2%	34.4%	38.3%	
Schooling (full years)					p< 0.001
	0-4	2.2%	6.3%	5.6%	
	5-10	20.4%	40.6%	37.0%	
	11 and over	77.4%	53.2%	57.4%	
	Mean (standard deviation)	11.5 (3.1)	9.8 (3.4)	10.1 (3.4)	
Number of prenatal care appointments					p< 0.001
	0	0.0%	1.2%	1.0%	
	1-5	4.0%	13.6%	12.0%	
	6 and over	96.0%	85.2%	87.0%	
	Mean (standard deviation)	9.4 (2.7)	8.6 (3.3)	8.7 (3.2)	
Trimester of prenatal care beginning (n=5.666)					p< 0.001
	First	90.0%	81.5%	83.0%	
	Second	9.6%	17.0%	15.7%	
	Third	0.4%	1.5%	1.3%	
Prenatal care type (n=5.666)					p< 0.001
	Public	11.6%	50.4%	43.6%	
	Private	88.4%	49.6%	56.4%	
Childbirth service used					< 0.001
	Public	16.8%	67.8%	59.0%	
	Private	83.2%	32.2%	41.0%	
Total		17.2% (985)	82.8%(4.736)	100% (5.721)	

The cesarean rate increased 107% between 2007 and 2016, from 10.5% (95%CI: 8.9%–12.2%) to 21.7% (95%CI: 19.5%–23.8%), as shown in Table 2. When analyzing this event per household income for the same period, a significant increase was observed among those belonging to the lowest income tertile, from 4.5% to 11.5%. This increase was also significant regarding schooling. On-demand cesarean sections for all categories studied practically doubled between 2007 and 2016. All these differences were significant (Table 2).

**Table 2:** On-demand cesarean section according to the year of the survey, household monthly income and maternal schooling. Rio Grande (RS), Brazil, 2007, 2010, 2013 and 2016. (n=5,721)

Variable		On-demand cesarean section % (n)				p-value *
		2007	2010	2013	2016	
On-demand cesarean section prevalence		10.5 (138)	18.2 (244)	17.8 (290)	21.7 (313)	< 0.001
Household monthly income (tertile)						
	1 (lower)	4.5 (29)	15.8 (85)	8.4 (16)	11.5 (16)	0.001
	2	13.0 (58)	16.1 (78)	12.1 (78)	13.9 (62)	0.757
	3 (higher)	23.1 (51)	25.4 (81)	24.8 (196)	27.3 (235)	0.183
Linear trend p-value		< 0.001	0.001	< 0.001	< 0.001	
Schooling (full years)						
	0-8	4.5 (22)	9.6 (46)	7.9 (40)	10.3 (40)	0.005
	9-11	12.8 (80)	22.5 (150)	18.6 (146)	22.2 (136)	0.001
	12 and over	18.9 (36)	24.9 (48)	30.9 (104)	30.8 (137)	0.002
Linear trend p-value		< 0.001	< 0.001	< 0.001	< 0.001	

* linear trend test for the period.

Table 3 shows that the absolute inequality (SII) in the performance of cesarean sections regarding household income increased by 4 p.p. (17%), from 24% in 2007 to 28% in 2016. This difference was even more evident in relation to schooling, which increased by 9 p.p. (48%), from 19% to 28%. Regarding relative inequality (RII), the odds of women in the largest-income tertile undergoing an on-demand cesarean section decreased 2.5 times, from 9.22 in 2007 to 3.73 in 2016. Regarding schooling, odds downwards slope was lower, 1.6 times, decreasing from 5.75 in 2007 to 3.66 in 2016.

**Table 3:** Measures of inequality in on-demand cesarean section in relation to parturients’ schooling and income in the four years of the study. Rio Grande (RS), Brazil. (n=5,721)

Indices	2007	2010	2013	2016
	**Household monthly income**			
SII (95%CI)*	0.24 (0.18–0.31)	0.12 (0.04–0.19)	0.27 (0.19–0.34)	0.28 (0.20–0.37)
RII (95%CI)**	9.22 (4.04–14.4)	1.86 (1.10–2.63)	4.38 (2.59–6.17)	3.73 (2.19–5.27)
	**Schooling (full years)**			
SII (95%CI)*	0.19 (0.13–0.26)	0.23 (0.16–0.31)	0.30 (0.23–0.37)	0.28 (0.21–0.35)
RII (95%CI)**	5.75 (2.61–8.88)	3.53 (2.11–4.94)	5.37 (3.29–7.44)	3.66 (2.38–4.95)

* SII: Slope index of inequality: absolute difference in health status between those at the bottom and those at the top of the income hierarchy

** RII: Relative index of inequality: odds ratio for each outcome comparing those at the bottom with those at the top of the income hierarchy

## DISCUSSION

The number of puerperae who underwent on-demand cesarean section in Rio Grande doubled between 2007 and 2016. Those belonging to higher-income households and who had better schooling were more commonly attended in their desire to perform cesarean. In this same period, absolute inequality increased and relative inequality decreased both for parturient household income and schooling.

The data shown here agree with on-demand cesarean section rates in other countries^4^. A recent review on on-demand cesarean section found a variation that ranged from 0.2% from all deliveries in Ireland to 24.7% in China^14^. Low-income countries, despite the scarce information available for this outcome, the substantial low quality of the obstetric care led women to demand cesarean sections without medical prescription. In a study in Niger, 39.6% of the women alleged that decided for cesarean section due to the low quality of the medical care, as peridural anesthesia and labor monitoring are widely unavailable^15^. However, the increase of on-demand cesarean section is also observed in medium and high-income countries^14^. Considering the studies that evaluated the occurrence of on-demand cesarean sections over time, an overall tendency of increase in its occurrence is observed. A study conducted in Switzerland between 2002 and 2008 showed an increased percentage of women who underwent on-demand cesarean section in this period, from 4.2% to 10.2%^16^. A study conducted in China found a marked increase in this occurrence between 1994 and 2006, from 0.4% to 20%^17^. In these countries, the main motivation was fear of vaginal delivery^14^.

Estimates for on-demand cesarean section are scarce in Brazil, and to this moment, there are no population-based studies to monitor its occurrence. National estimates can be traced through the second edition of the Birth in Brazil: national survey into labor and birth. In the first edition of this hospital-based study, performed in 2011-2012, the prevalence of on-demand cesarean section was of 9.9% among primiparae and of 12.4% among multiparae, considering all deliveries. It was also predominant in the private health system, reaching up to 27.1% among primiparae and 31.1% among multiparae. Fear of vaginal delivery was the main reason underlying the decision for cesarean section, especially among the primiparous women^3^.

The increased rate of on-demand cesarean section observed in our study may be partly explained by the economic transformation the municipality suffered in the last 10 years. In the period 2010-2017, Rio Grande experienced the rise of the Naval Complex, with peaks in 2013 and 2014, when it created more than 7,400 direct jobs, resulting in significant income gains and access to private health plans, which were borne by companies^18^. On-demand cesarean section is greater in the private system because doctors are more likely to fulfil the parturient's desire^19^.

At least 83.0% of women who underwent on-demand cesarean sections in our study were treated in the private system, both in prenatal care and delivery. Cesarean section is increasingly associated with a product that the patient acquires as a sign of social or economic status^8^. Any cesarean section performed without proper medical indication can be considered unnecessary and, therefore, at risk of bringing more harm than benefits to women and, above all, newborns^7^.

The prevalence of on-demand cesarean section was slightly higher in 2010 when compared with 2013. The study of 2010 showed the highest proportion of on-demand cesarean sections among women of the lowest-income terciles in comparison with the other surveys, with highest prevalence found among the women with higher income and schooling.

Higher-income and better-schooled puerperae were more commonly attended in their desire to perform the cesarean section. This reinforces the idea that the choice of delivery type is based much more on purchasing power than on clinical indication, which is unsettling, since these patients are the ones with the lowest obstetric risk^19^. Detailed studies involving a qualitative approach show that physicians are less likely to refuse the request of women with better schooling and with greater argumentative power^20^^,^^21^.

It is also noteworthy that women with lower household income and worse schooling have the greatest increase in the proportion of on-demand cesarean sections during this period. Poorest mothers seem to reproduce the behavior of mothers of better socioeconomic levels, which is well documented by studies on the occurrence of cesarean sections conducted in the Pelotas cohorts^8^^,^^20^.

This can be supported by the false idea that the medical intervention implies increased attention during the procedure, creating a market for unnecessary interventions among women that feel that the medical technology is inaccessible to them^20^.

This increased rate of on-demand cesarean sections among women from lower socioeconomic strata was also responsible for the decreased relative inequality in the period. Obviously, reduced inequality in these circumstances is not what is desired. The ideal would be to control on-demand cesarean section in all categories, especially in those with a better socioeconomic level, in which this prevalence is higher. In Rio Grande, lower levels of inequality were a consequence of higher levels of this procedure in the worst socioeconomic groups. Regarding absolute inequality, the gap of this outcome between extreme income and schooling groups caused its increase, suggesting a greater gap between the groups. This implies that these analyses of inequality showed that odds of worse socioeconomic level puerperae being submitted to cesarean section increased and that gap from the best level group was even wider because outcome in this group also increased significantly. It suggests that improved schooling and household income observed in this municipality over the years could not promote the appropriate use of health services. On the contrary, we observed an increased access to a technology that exposes mothers and newborns to an unnecessary procedure that can be harmful. However, this inadequate consumption of health services tends to equate the care of the poorest mothers with the richest ones, which seems to be a desire of the first group, as already reported in a study conducted in the southern region of Brazil^8^^,^^20^.

We believe the construction of the outcome was based exclusively on the parturient's report when interpreting the results. Thus, it cannot be affirmed that pregnant women that chose cesarean sections had no clinical need. Moreover, all those who underwent this procedure had their request agreed by the doctor. This is a very important point that must be debated by the scientific society, the public prosecutor's office and health professionals involved in prenatal care and, especially, in childbirth care. Mothers must be instructed about the risks of this procedure. On-demand cesarean sections will increasingly be one of the most important components in determining overall cesarean rates without the commitment of health professionals and laws that inhibit or at least regulate this practice^4^.

It is noteworthy that in 2016, although the Federal Council of Medicine and the Ministry of Health legitimize women's wish for the type of delivery, they have established the minimum condition for pregnancy of at least 39 weeks and that mothers sign a term indicating they are aware of the risks and benefits of this choice. Some impact on cesarean sections is expected in the country toward reducing the performance of on-demand cesarean sections.

The increased on-demand cesarean sections in the study location is unsettling, despite the decreasing gap between extreme categories as a consequence of higher levels of on-demand cesarean sections among women of lower income and worse schooling. In addition to studies with more robust designs such as cohort studies, we think those using a strong qualitative component are also necessary to further understand the reasons that cause mothers to undergo a procedure, sometimes unnecessary, which can entail risks to the mother and child, and also to the doctor for accepting its performance without evidenced clinical need.
